# *Pasteurella multocida* from deep nasal swabs and tracheobronchial lavage in bovine calves from Sweden

**DOI:** 10.1186/s13028-024-00781-7

**Published:** 2024-11-05

**Authors:** Mattias Myrenås, Märit Pringle, Boel Harbom, Björn Bengtsson

**Affiliations:** Department of Animal Health and Antimicrobial Strategies, Swedish Veterinary Agency, 751 89 Uppsala, Sweden

**Keywords:** Bovine respiratory disease, Cattle, cgMLST, MLST

## Abstract

**Background:**

Bovine respiratory disease (BRD) is common in intensively raised cattle and is often treated with antibiotics. For practitioners, knowledge of the bacteria involved in an outbreak and their antibiotic susceptibility is warranted. To this end, samples from the upper or lower respiratory tract of calves can be submitted for bacteriological culture and susceptibility testing of relevant isolates. However, it is debated whether isolates from the upper respiratory tract are representative of bacteria causing infections in the lower respiratory tract. In this study, we used MALDI-TOF MS, multilocus sequence typing (MLST) and core-genome multilocus sequence typing (cgMLST) to compare culture results of 219 paired samples (sample pairs) of deep nasal swabs (DNS) and tracheobronchial lavage (TBL). The sample pairs came from 171 calves in 30 calf groups across 25 farms with 48 calves sampled twice.

**Results:**

The predominant bacterial pathogen was *Pasteurella multocida*, which was isolated from 37.4% of DNS and 22.4% of TBL. There was no statistically significant difference in isolation frequency of *P. multocida* between calves considered healthy and those suspected for BRD for DNS (*P* = 0.778) or TBL (*P* = 0.410). Among the 49 sample pairs where *P. multocida* was isolated from TBL, the same species was isolated from DNS in 29 sample pairs (59.2%). Isolates from 28 of these sample pairs were evaluated by MLST, and in 24 pairs (86.0%) *P. multocida* from DNS and TBL were of the same sequence type (ST). Moreover, cgMLST showed that the genetic distance between isolates within 21 of the 28 sample pairs (75.0%), was less than two alleles, and DNS and TBL isolates were considered identical. In seven sample pairs (25%), the genetic distance was greater, and DNS and TBL isolates were considered nonidentical.

**Conclusions:**

*Pasteurella multocida* was readily isolated from DNS and in calves where this species was isolated also from TBL, DNS and TBL isolates were identical in 75% of the sample pairs. This suggests that during an outbreak of BRD, submission of DNS samples from 4 to 6 calves could be a convenient approach for practitioners seeking guidance on *P. multocida* present in the lower respiratory tract and their antibiotic susceptibility.

**Supplementary Information:**

The online version contains supplementary material available at 10.1186/s13028-024-00781-7.

## Background

Bovine respiratory disease (BRD) is common in intensively raised cattle and represents a major economic and health problem in cattle herds worldwide [1–5]. The background of BRD is multifactorial and includes infectious agents and predisposing factors related to the immunological and general status of the animals as well as to management and housing [6–8]. Due to its multifactorial background, the pathogenesis of BRD varies, but in general, viral infections of the upper respiratory tract precede bacterial infections of the lower respiratory tract [9]. The bacteria commonly involved often reside in the upper airways of healthy calves and include *Pasteurella multocida*, *Mannheimia haemolytica*, *Histophilus somni* and *Mycoplasmopsis bovis* [8–10].

The control of BRD is complex and involves measures to hinder the spread of infectious agents and mitigate predisposing factors [7, 11–13]. Therapeutic, prophylactic or metaphylactic antibiotic treatments are also used to control BRD, but routine use of antibiotics should be avoided due to the risk of emerging antibiotic resistance [6, 11, 12, 14]. For reasons of animal welfare and to minimise economic losses, however, it is often necessary to use therapeutic antibiotic treatments when BRD occurs in a group of animals [11].

Considering the increased occurrence of acquired antibiotic resistance, it is important that treatment is guided by knowledge of the antibiotic susceptibility of the relevant bacteria [5, 15–18]. Information on the antibiotic susceptibility of bacteria involved in BRD is available from national monitoring programs, e.g., [19–24], and from other scientific literature [16, 25–27]. However, such compiled data are derived from sources with varying study designs and from different farm types and might not be representative of the situation on a specific farm [18]. It is therefore preferable if information is available for relevant bacteria isolated from acutely affected and untreated animals from the farm where treatments are to be instituted [18]. This is even more relevant for farms experiencing treatment failures where the presence of antibiotic resistance could cause re-evaluation of treatment protocols [28]. Moreover, in some countries, susceptibility testing of isolates from animals on a farm is mandatory for the use of specific antibiotics on that farm [16, 29].

To obtain isolates of bacterial respiratory pathogens for susceptibility testing, it is convenient and simple for practitioners to collect samples for bacteriological culture from the upper respiratory tract using nasal swabs (NS), deep nasal swabs (DNS) or nasopharyngeal swabs (NPS) [5, 28]. Samples from the lower respiratory tract can also be collected in a clinical setting. Several different techniques are available, including bronchoalveolar lavage (BAL), tracheobronchial lavage (TBL), transtracheal wash (TTW) or transtracheal swab (TTS) [5, 28, 30]. These methods are, however, more complicated, and invasive than NS, DNS or NPS and are considered more stressful for the animals [15, 25, 31].

To collect samples from the upper respiratory tract is convenient, but it is debated whether isolates from NS, DNS or NPS are representative of bacterial respiratory pathogens in the lower respiratory tract, which are the targets for antibiotic therapy [5, 18, 32]. However, in studies that have compared paired isolates of *Pasteurellacae* from the upper and lower respiratory tracts of calves, there is generally moderate to almost perfect agreement at the species level [15, 28, 29, 33–35]. For *M. haemolytica*, there was also a high agreement between paired isolates from the upper and lower respiratory tract when molecular methods were used to compare isolates [15, 35, 36]. However paired isolates of *P. multocida* from the upper and lower respiratory tracts of calves have not been compared by molecular methods.

Therefore, the aim of this study was to evaluate whether DNS samples from the upper respiratory tract of calves provide comparable information regarding bacterial pathogens in TBL samples from the lower respiratory tract of the same calves. Our hypothesis is that bacterial pathogens in the lower respiratory tract of a calf can generally also be cultured from DNS from the same calf or from other calves in the same group. To test this hypothesis, we used data from field investigations and reanalysed a collection of strains from the investigations using matrix-assisted laser-desorption ionisation-time-of-flight (MALDI-TOF MS), multilocus sequence typing (MLST) and core-genome multilocus sequence typing (cgMLST). MLST is a method, first described and evaluated for *Neisseria meningitidis*, in which the gene sequences of, usually, seven housekeeping genes are compared [37]. The number of identical gene variants in an isolate-to-isolate comparison makes it possible to calculate the evolutionary relationships between isolates of the same species. The cgMLST also compares gene variants obtained from genome analysis but the genome sequences enable the comparison of 1609 genes instead of 7. Hence, cgMLST can show relatedness between isolates with considerably higher detail than MLST.

## Methods

### Field investigations

The data and bacterial isolates in this study are from field investigations from 1997 to 2000 on Swedish cattle farms experiencing outbreaks of BRD and affiliated with a health control program through the Swedish Animal Health Service [38]. To manage the outbreaks, the calves were examined clinically, and those with a rectal temperature > 39.5 °C and at least one of the clinical signs of nasal discharge, cough or abnormal respiratory sounds on auscultation, were considered suspected for BRD. To assess antibiotic susceptibility of possible bacterial respiratory pathogens, 3–6 calves from a calf group on farms where BRD occurred were sampled at each visit. A calf group was defined as a group of calves confined in the same or adjacent pens on a farm. The size of calf groups varied between farms from about 15 to 100 calves. When only a few calves suspected for BRD were available, calves considered healthy were sampled instead. Samples were collected from the posterior nasal cavity (called DNS samples) using cotton swabs (Amies charcoal media, Copan Diagnostics, Brescia, Italy). The swab was entered into the nasal cavity to its full length (≈12 cm) after dry cleaning of the outer nares with medical cotton. Bacteriological samples were also collected from the lower respiratory tract by tracheobronchial lavage (TBL). TBL was collected as previously described using a catheter designed to protect the sample from contamination when passed through the nasal cavity [30]. In brief, after dry cleaning of the outer nares with medical cotton, a catheter made of silastic tubing (diameter 5 mm, length 50 cm) with an inner lining of teflon tubing was passed through the nasal cavity into the trachea. A silicon rubber stopper 10 cm from the upper end of the catheter hindered passage more than 40 cm into the airways. When located in the trachea, a 100 cm flexible teflon tube was passed through the catheter and through a slit in a silicon tip sealing the catheter. The teflon tube was then passed down to the region of the tracheal bifurcation where 20 mL of isotonic saline was instilled and immediately aspirated. The mean volume of fluid retracted as a sample was 4.5 mL (range 0.5–15 mL).

In all, 37 different farms were visited, 400 calves were examined and sampled, and 80 of the calves were examined and sampled again within 3–9 weeks. The DNS and TBL samples collected from a calf at the same time is hereafter referred to as a “sample pair” and the isolates cultured from a sample pair referred to as an “isolate pair”.

The DNS and TBL samples collected were kept at + 8 °C and transported within 24 h to the National Veterinary Agency, Sweden (SVA), where they were cultured for respiratory bacterial pathogens on blood agar, blue agar and hematin agar (SVA, Uppsala, Sweden) at 37 °C overnight. Colonies with a macroscopic appearance in agreement with *Pasteurellacae* were subcultured and identified by biochemical tests. The culture results were registered as “Pasteurella-like bacteria” (PLB), “Other bacteria” (OB) or “Negative culture” (NEG).

Descriptive data for farms and individual calves from the field investigations were stored together with laboratory results. Isolates identified as PLB were stored at −80 °C, and isolates identified as OB were not stored.

As the calves were examined and sampled in the context of the routines for managing clinical outbreaks of BRD, and not for experimental purposes, no ethics approval was sought for.

### Selection of calves from the field investigations

From the field investigations involving 400 calves, those selected for the present study met the following criteria: (1) both DNS and TBL should have been collected; (2) no antibiotic treatment within 3 days prior to sampling; (3) data should be available for at least 3 calves in the calf group; (4) isolates identified as PLB should be available in the strain collection.

Using above criteria, 219 sample pairs from 171 different calves were selected for this study. The calves were from 30 different calf groups on 25 farms. Twelve calf groups on ten farms had been visited twice and 48 individual calves from 11 calf groups on nine farms had been sampled twice. Ninety of the sample pairs were from calves suspected for BRD and 129 sample pairs from calves considered healthy (Table 1, Supplementary Table 1).

**Table 1:** Descriptive data from field investigations in 1997–2000

Farm ID	Type^a^	Calf group	Sampling occasion 1		Interval S1–S2 (days)	Sampling occasion 2		Number of calves sampled:		Total number of DNS/TBL sample pairs^d^
			Number of calves^b^	Age (days) Mean (range)		Number of calves	Age (days) Mean (range)	Once	Twice	
1	Meat	A	6 (1)	61 (19–171)				6	0	6
2	Meat	A	4 (2)	NA^c^	33	4 (3)		0	4	8
		B	4 (4)	NA				4	0	4
		C	6 (4)	NA	43	4 (1)		2	4	10
5	Meat	A	6 (3)	80 (63–130)				6	0	6
6	Meat	A	5 (2)	85 (46–129)	35	4 (1)	127 (81–164)	5	2	9
		B	6 (0)	95 (76–131)				6	0	6
7	Meat	A	6 (3)	98 (84–113)	29	4 (0)	131 (123–143)	2	4	10
9	Meat	A	3 (0)	118 (94–154)	59	3 (1)	NA	6	0	6
10	Meat	A	4 (1)	88 (58–115)	49	5 (3)	126 (107–159)	3	3	9
		B	6 (2)	NA	49	5 (0)	NA	1	5	11
11	Meat	A	4 (2)	55 (49–58)	34	4 (2)	89 (84–93)	2	3	8
		B	5 (3)	51 (43–59)				5	0	5
13	Meat	A	6 (3)	NA	33	6 (0)	NA	0	6	12
15	Meat	A	6 (3)	NA				6	0	6
16	Meat	A	6 (4)	102 (79–128)				6	0	6
17	Dairy	A	6 (4)	NA				6	0	6
18	Meat	A	5 (4)	92 (72–112)				5	0	5
22	Meat	A	5 (1)	99 (83–124	32	6 (1)	139 (123–164)	1	5	11
25	Meat	A	6 (2)	103 (92–130)				6	0	6
28	Meat	A	5 (3)	98 (86–113)				5	0	5
29	Dairy	A	5 (2)	55 (20–79)				5	0	5
31	Meat	A	6 (4)	67 (44–116)				6	0	6
33	Meat	A	5 (3)	78 (62–91)				5	0	5
34	Meat	A	6 (4)	102 (77–134)				6	0	6
35	Meat	A	6 (6)	NA				6	0	6
36	Meat	A	6 (2)	60 (43–97)				6	0	6
40	Dairy	A	6 (1)	52 (19–73)	29	6 (2)	81 (48–102)	0	6	12
44	Meat	A	6 (1)	77 (73–86)				6	0	6
46	Dairy	A	6 (2)	60 (25–83)	20	6 (0)	80 (45–103)	0	6	12
Total			162 (76)	80 (19–171)		57 (14)	108 (45–164)	123	48	219

Data for the 171 different calves from 30 calf groups on 25 farms selected for the present study. Ten farms were visited twice and 48 calves from 11 calf groups on nine farms were sampled two times on separate occasions

^a^Meat = farms specialised in raising calves for meat production, Dairy = farms with milk production

^b^Number of calves suspected for BRD in parenthesis

^c^Data not available

^d^A sample pair is the DNS and TBL samples collected from the same calf at the same time

### Selection of isolates for species identification by MALDI-TOF

To refine the identification of bacterial species, isolates identified as PLB in the field investigations were analysed by MALDI-TOF MS. From the calves selected for this study 136 isolates (85 from DNS and 51 from TBL) from 104 sample pairs were available in the strain collection. In 32 sample pairs isolates were available from both DNS and TBL, whereas in 72 isolates pairs isolates were available either from DNS (53 isolates) or from TBL (19 isolates). All available isolates were analysed by the MALDI Biotyper system (Bruker Daltonics, Bremen, Germany) to identify the species. Material from a single colony from the agar plate was spotted on a MALDI plate without pretreatment. The spots were covered with 1 µL of matrix solution consisting of α-cyano-4-hydroxycinnamic acid (HCCA), air-dried at room temperature, and introduced into the MALDI-TOF mass spectrometer for analysis. The spectra of all the isolates were compared to the spectra in the database, and identification was provided with a reliability score. A score ≥ 2.0 was considered reliable for species identification.

### Whole-genome sequencing and genome assembly

In 29 sample pairs, *P. multocida* was isolated from both DNS and TBL and the genomes of DNS and TBL isolates from 28 of these sample pairs (56 isolates from 25 different calves) were sequenced and compared (isolates from one sample pair were not available) (Table 4). Colony material was collected from blood agar plates for DNA extraction using a Qiagen EZ1 DNA Tissue Kit (Qiagen, Halden, Germany). Nextera Library preparation (Illumina, Foster City, United States) and paired-end sequencing (2 × 150 bp) was performed at Clinical Genomics Stockholm, SciLifeLab (Solna, Sweden) using an Illumina NovaSeq 6000 instrument. The raw reads for each sample were quality checked using FastQC vo.11.9 [39], trimmed using Trimmomatic v0.39 [40] and assembled using SPAdes v3.14.0 [41]. The assemblies were error-corrected using Pilon v1.23 [42]. Details on the analysis parameters and FastQC data can be found in Supplementary Table 2.

### Multilocus sequence typing

For *P. multocida*, there are two public MLST schemes [43, 44] available at PubMLST [45], of which we chose to use the multihost MLST scheme previously described [43].

### Core-Genome multilocus sequence typing and minimum spanning tree construction

No cgMLST schemes for *P. multocida* suitable for global nomenclature are currently available. Therefore, we constructed an ad hoc cgMLST scheme from the project data suitable for analysis of this dataset alone using the cgMLST Target Definer v.1.5 function in SeqSphere + version 6.0.2 (Ridom, Würzburg, Germany). We used the annotated genome with the GenBank accession number NZ_CP008918.1 as the seed genome and the genomes NZ_CP015569.1, NZ_CP037861.1 and NZ_CP037865.1 as the penetration genomes and excluded hits found in the plasmid sequence NC_017035.1, gene duplicates and truncated genes. This resulted in a cgMLST scheme consisting of 1611 targets, as documented in Supplementary Table 1. Using the results from 1609 of the cgMLST targets, the phylogenetic distance was calculated using the Minimum Spanning Tree method (Kruskal JB 1956) using GrapeTree v.1.5.0 [46].

### Statistical calculations

Differences in culture results between DNS and TBL were evaluated by Fischer’s exact test, with *P* ≤ 0.05 considered evidence of a significant difference. Agreement between culture results for DNS and TBL for *P. multocida* was evaluated by Kappa statistics and McNemar’s test [47]. Kappa values (ƙ) were interpreted to indicate the strength of agreement as follows: < 0.20, slight; 0.20–0.40, fair; 0.41–0.60, moderate; 0.61–0.8, substantial; and > 0.8, almost perfect. The exact McNemar significance probability test was used to determine the potential for bias between DNS and TBL, and values ≤ 0.05 were considered evidence of bias. Statistical calculations were made using the GraphPad software.

## Results

### Bacteriological culture

Among the 219 DNS samples, *P. multocida* was isolated from 82 (37.4%), *M. haemolytica* from one (0.5%), *Moraxella bovoculi* from two (0.9%) and other bacteria (OB) from 134 samples (61.2%) (Table 2). Among bacteria assigned as OB in this study one isolate originally diagnosed as *Trueperella pyogenes* is included (Supplementary Table 1). In DNS there was no difference in the isolation frequency of *P. multocida* between calves considered healthy (38.0%) and those suspected for BRD (36.7%) (*P* = 0.778). Among the 219 TBL samples, *P. multocida* was isolated from 49 (22.4%), *M. bovoculi* from two (0.9%), and OB from 33 (15.1%), whereas most samples, 135 (61.6%), yielded no growth. Among bacteria assigned as OB in this study three isolates originally diagnosed as *Trueperella pyogenes* were included (Supplementary Table 1). In TBL there was no difference in the isolation frequency of *P. multocida* between calves considered healthy (20.2%) and those suspected for BRD (25.6%) (*P* = 0.410), but the proportion of negative cultures was greater in calves considered healthy (68.2%) than in those suspected for BRD (52.2%) (*P* = 0.023).

**Table 2:** Crosstabulation of culture results for deep nasal swabs (DNS) and tracheobronchial lavage fluid (TBL)

		TBL				Total
		*P. multocida*	*M. bovoculi*	Other bacteria	Negative culture	
DNS	*P. multocida*	29 (13.2%)		7 (3.2%)	46 (21.0%)	82 (37.4%)
	*M. haemolytica*	1 (0.5%)				1 (0.5%)
	*M. bovoculi*		2 (0.9%)			2 (0.9%)
	Other bacteria	19 (11.9%)		26 (11.9%)	89 (40.6%)	134 (61.2%)
	Total	49 (22.4%)	2 (0.9%)	33 (15.1%)	135 (61.6%)	219 (100%)

Data for 219 sample pairs from 171 different calves, 48 calves were sampled twice

Crosstabulation of culture results for the 219 sample pairs of DNS and TBL showed that when *P. multocida* or *M. bovoculi* were isolated from TBL, the same species were isolated from DNS in 60.8% (31/51) of the sample pairs (Table 2). In the remaining 20 sample pairs where *P. multocida* was isolated from TBL, *M. haemolytica* was isolated from DNS in one sample pair and OB in 19 sample pairs. Conversely, when *P. multocida* or *M. bovoculi* were isolated from DNS, the same species was isolated from TBL in 36.9% (31/84) of the sample pairs (Table 2). In the remaining 53 sample pairs where *P. multocida* was isolated from DNS, the TBL samples were negative in 46 sample pairs and yielded OB in seven sample pairs. The agreement between DNS and TBL culture results for *P. multocida* was slight for calves considered healthy, with a possible bias in the dataset (ƙ 0.14 ± 0.16, McNemar *P* = 0.001), and fair for calves suspected for BRD, with no bias in the dataset (ƙ 0.34 ± 0.20, McNemar *P* = 0.078).

Out of 42 sampling times across different farms at the two sampling occasions, *P. multocida* was isolated from DNS in 35 (83.3%) of these sampling times within the 30 calf groups and in at least one calf per sample group, with a median isolation frequency of 33.0% (range: 0–100%). From TBL, *P. multocida* was isolated from at least one calf in the sampled group at 31 (73.8%) of the 42 sampling times within the 30 different calf groups and the median isolation frequency was 23.0% (range: 0–100%) (Table 3).

**Table 3:** Cross tabulation within calf groups and sampling occasions of culture results

Farm ID	Calf group	Sampling occasion	DNS	TBL				Total	Isolation frequency of *P. multocida*	
				*P. multocida*	*M. bovoculi*	Other	Negative		DNS	TBL
1	A	1	*P. multocida*				1	1	17%	17%
			Other	1		1	3	5		
2	A	1	*P. multocida*	1				1	25%	25%
			*M. bovoculi*		2			2		
			Other			1		1		
		2	*P. multocida*	1		1		2	50%	25%
			Other				2	2		
	B	1	*P. multocida*	1		1		2	50%	25%
			Other			2		2		
	C	1	*P. multocida*	1		1	2	4	67%	17%
			Other				2	2		
		2	*P. multocida*				1	1	25%	25%
			Other	1			2	3		
5	A	1	*P. multocida*	3				3	50%	50%
			Other			1	2	3		
6	A	1	*P. multocida*	2			1	3	60%	60%
			*M. haemolytica*	1				1		
			Other				1	1		
	B	1	*P. multocida*	1			2	3	75%	25%
			Other				1	1		
		2	*P. multocida*				1	1	17%	0%
			Other				5	5		
7	A	1	*P. multocida*	2			2	4	67%	33%
			Other				2	2		
		2	*P. multocida*	1			2	3	75%	25%
			Other				1	1		
9	A	1	*P. multocida*				1	1	33%	0%
			Other				2	2		
		2	*P. multocida*	1			1	2	67%	33%
			Other			1		1		
10	A	1	*P. multocida*	1			3	4	100%	25%
		2	Other	1		1	3	5	0%	20%
	B	1	*P. multocida*	1			1	2	33%	33%
			Other	1		2	1	4		
		2	*P. multocida*	1			1	2	40%	40%
			Other	1			2	3		
11	A	1	*P. multocida*	2			1	3	75%	75%
			Other	1				1		
		2	*P. multocida*	2			2	4	100%	50%
	B	1	*P. multocida*	2			1	3	60%	40%
			Other			1	1	2		
13	A	1	*P. multocida*			1		1	17%	50%
			Other	3		1	1	5		
		2	*P. multocida*				1	1	17%	0%
			Other			2	3	5		
15	A	1	Other	1		2	3	6	0%	17%
16	A	1	Other			1	5	6	0%	0%
17	A	1	Other	2		1	3	6	0%	33%
18	A	1	Other			2	3	5	0%	0%
22	A	1	*P. multocida*				2	2	40%	0%
			Other				3	3		
		2	*P. multocida*				1	1	17%	17%
			Other	1		2	2	5		
25	A	1	*P. multocida*				1	1	17%	0%
			Other			2	3	5		
28	A	1	*P. multocida*	3			1	4	80%	60%
			Other				1	1		
29	A	1	*P. multocida*			1		1	20%	0%
			Other			1	3	4		
31	A	1	Other	1			5	6	0%	17%
33	A	1	*P. multocida*				1	1	20%	0%
			Other				4	4		
34	A	1	*P. multocida*				2	2	33%	17%
			Other	1			3	4		
35	A	1	*P. multocida*	2				2	33%	33%
			Other				4	4		
36	A	1	*P. multocida*				2	2	33%	17%
			Other	1			3	4		
40	A	1	*P. multocida*	1		1	2	4	67%	17%
			Other				2	2		
		2	*P. multocida*			1	3	4	67%	0%
			Other			1	1	2		
44	A	1	Other				6	6	0%	0%
46	A	1	*P. multocida*				4	4	67%	17%
			Other	1		1		2		
		2	*P. multocida*				3	3	50%	33%
			Other	2			1	3		
			Total	49	2	33	135	219	37%	22%

Data for deep nasal swabs (DNS) and tracheobronchial lavage fluid (TBL) from 171 calves (48 calves sampled twice) and 30 calf groups (12 groups sampled twice)

### Genotyping

**Fig. 1:**
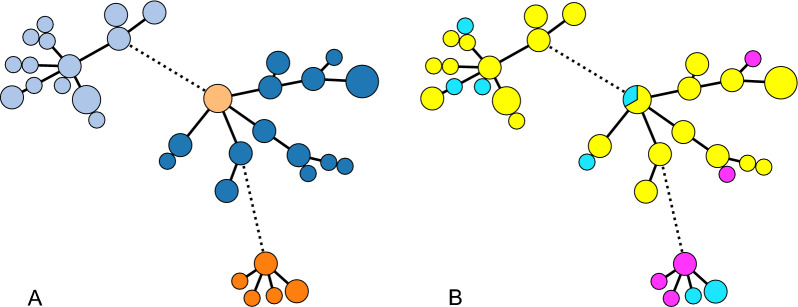
Minimum spanning for *P. multocida* isolates. Minimum spanning tree constructed from core genome multilocus sequence typing data consisting of 1609 loci showing 56 *P. multocida* isolates from deep nasal swabs (DNS) and tracheobronchial lavage (TBL). Panel **A**: ST-3 is shown in light blue, ST-19 in dark orange, ST-68 in dark blue and ST-202 in light orange. Panel **B**: cgMLST differences between DNS and TBL sampled on the same occasion from the same calf. Pairs of identical isolates (0 or 1 allele difference from 1609) are coloured yellow, related isolates (26 to 74 alleles) are coloured magenta, and nonrelated isolates (1041 to 1443 allele difference) are coloured cyan. The circle size indicates the number of isolates. Branches are dotted when there are differences in alleles greater than 200

Among the 56 isolates from the 28 isolate pairs where *P. multocida* was isolated from both DNS and TBL, four different MLST sequence types (ST) were found (Table 4; Fig. 1A). Twenty-five isolates were ST-68, 21 were ST-3, and seven were ST-19. Three isolates were of a ST which was reported to the Public databases for molecular typing and microbial genome diversity (PubMLST) and named ST-202. In 24 of the 28 isolate pairs (85.7%), *P. multocida* from DNS and TBL had the same STs, whereas in four isolate pairs (14.3%), the STs of the DNS and TBL isolates differed (Table 4, Fig. 1B). In the three calves that were sampled twice, *P. multocida* of the same ST was isolated on sampling occasion 1 and 2 from one calf, whereas there was a shift in STs between sampling occasion 1 and 2 in two calves (Table 4).

**Table 4:** Sequence types (STs) of *P. multocida*

Farm ID	Calf group	Calf ID	Sequence types			
			Sampling occasion 1		Sampling occasion 2	
			DNS	TBL	DNS	TBL
2	A	950			ST-3	ST-3
		951	ST-68	ST-68		
	B	960	ST-3	ST-3		
	C	35	ST-68	ST-68		
5	A	1737	ST-68	ST-68		
		1739	ST-68	ST-68		
		1749	ST-3	ST-19		
6	A	428	ST-68	ST-68		
		444	ST-68	ST-68	ST-3	ST-3
7	A	1016	ST-19	ST-3		
		2039	ST-19	ST-202	ST-202	ST-202
9	A	5062	ST-3	ST-3		
10	A	9205	ST-68	ST-68		
	B	9725	ST-68	ST-68	ST-68	ST-68
11	A	72	ST-19	ST-19		
		91	ST-19	ST-19		
		1011			ST-68	ST-68
		6051			ST-68	ST-68
	B	828	ST-3	ST-3		
28	A	1077	ST-68	ST-3		
		5079	ST-3	ST-3		
		7067	ST-68	ST-68		
35	A	25	ST-3	ST-3		
		30	ST-3	ST-3		
40	A	482	ST-3	ST-3		

Data for 28 isolate pairs from 25 calves (3 calves sampled twice) where isolates were available from both deep nasal swabs (DNS) and tracheobronchial lavage fluid (TBL)

In groups of calves and within farms, *P. multocida* of more than one ST was often isolated. Thus, at the seven sampling times where data were available for more than one calf in a group, *P. multocida* of a single ST was isolated from DNS and TBL from all calves at four sampling times (Farm 6, Calf group A, sampling occasion 1; Farm 11, Calf group A sampling occasion 1; Farm 11, Calf group A, sampling occasion 2; Farm 35, Calf group A, sampling occasion 1), whereas the isolates were of two or more STs at three sampling times (Table 4). Similarly, on the three farms (Farm 2, Farm 10, Farm 11) where more than one calf group was sampled, isolates of a single ST were found only on Farm 10 (Table 4).

The minimum spanning tree (MST) from the cgMLST data (Fig. 1A) showed that the *P. multocida* isolates clustered into three groups with closely related isolates. The genetic distance between DNS and TBL isolates within isolate pairs was 0 alleles in 17 isolate pairs and 1 allele in 4 isolate pairs. We interpret this as indicating that the *P. multocida* isolates within these pairs have a common ancestor in near-time and are examples of a limited within-host diversity which reflects the ongoing evolution of *P. multocida* into subpopulations. We group these isolates together as identical or near identical and this is the case in 21 of the 28 isolate pairs (75%) (Fig. 1B). In 3 isolate pairs, the genetic distance was between 26 and 74 alleles, and in 4 isolate pairs, it was between 1041 and 1443 alleles (Fig. 1B). The isolates within these 7 pairs were considered non identical.

The data for this study have been deposited in the European Nucleotide Archive (ENA) at EMBL-EBI under accession number PRJEB73847 [48].

## Discussion

The aim of this study was to evaluate whether bacterial pathogens isolated from DNS are representative for those found in the lower respiratory tract of calves. Since *P. multocida* was the only relevant pathogen isolated from both DNS and TBL, only this species could be evaluated. The agreement for *P. multocida* between DNS and TBL samples in calves considered healthy was slight (ƙ 0.14), and the McNemar test (*P* = 0.001) indicated that the data were biased. A higher agreement (fair, ƙ 0.28) was previously reported for *P. multocida* from the upper and lower respiratory tract of healthy calves [29]. For calves suspected for BRD in our study, the agreement was fair (ƙ 0.34), and the McNemar test indicated an unbiased dataset (*P* = 0.078). A greater agreement can be expected for diseased calves since *P. multocida* is considered a commensal in calves [2, 25, 49], and its presence in the upper respiratory tract does not imply infection in the lower respiratory tract [2]. Thus, agreements ranging from moderate to almost perfect (ƙ 0.48- > 0.8) have been reported for calves with BRD [28, 29, 36] and also for a material including both healthy calves and calves with clinical signs of respiratory disease [33]. In these studies, moderate to almost perfect agreement was also reported for *M. haemolytica*, and slight to substantial agreement for *H. somni*. The generally lower agreement for isolation of *P. multocida* in DNS and TBL in our study than in those cited above could be caused by underdiagnosis of relevant bacterial pathogens in DNS and TBL or misclassification of the health status of calves, as discussed below.

Although an agreement at the species level between bacterial isolates from the upper and lower respiratory tract suggests that the same strain is isolated from both sites, this is not necessarily true. In this study, MLST showed that *P. multocida* were of the same ST within 24 of the 28 isolate pairs evaluated, whereas different STs were found within four isolates pairs. This indicates that although there was an agreement at the species level, the same strain was isolated from DNS and TBL in only 24 of the 28 sample pairs. However, even an agreement at the ST level does not ensure that the isolates are identical. In this study the more discriminatory cgMLST showed that in the 24 isolate pairs which agreed at the ST level, there was no difference in alleles within 17 isolate pairs, and a difference of one allele within four pairs. We considered this to confirm that the same strain of *P. multocida* was present in DNS and TBL in 21 (75%) of the 28 sample pairs evaluated. However, three isolate pairs with an agreement at the ST level, differed between 26 and 74 alleles on cgMLST and are most likely also closely related but not identical. In contrast, the four isolate pairs which disagreed at the ST level, differed by 1041 to 1443 alleles within pairs and it is evident that different strains of *P. multocida* were isolated from DNS and TBL in these sample pairs. There are no previous reports evaluating genotypes of *P. multocida* from the upper and lower respiratory tract. However, for *M. haemolytica* evaluated by molecular methods a high level of agreement between isolates from the upper and lower respiratory tract has also been reported [15, 35, 36].

The fact that the same *P. multocida* strain was isolated from the upper and lower respiratory tract in 75% of the sample pairs (DNS and TBL) suggests that isolates from DNS generally, but not always, reflect relevant information for lower respiratory tract infections in the same animal. However, within calf groups and farms, often *P. multocida* of more than one MLST ST were isolated and cgMLST revealed that even isolates of the same MLST ST were not always identical. This shows that more than one strain of *P. multocida* can be present in individual animals as well as in a group of calves and that the panorama of strains on a farm is variable. This agrees with other studies where MLST of *P. multocida* has shown that isolates with different STs can be present in a group of calves, although often one or two types predominate on a farm, [50–53] and it is proposed that the movement of animals may cause greater variability [60].

Relevant bacterial pathogens (*P. multocida* and *M. haemolytica*) were cultured from 37.9% of the DNS samples. *P. multocida* was highly predominant which confirms that this species can be readily isolated from the upper airways of both healthy calves and from calves suspected for BRD [2]. A wide range of isolation frequencies for *P. multocida* (20–70%) have previously been reported for calves sampled by DNS or NPS [15, 17, 25, 28, 29, 31, 33, 36, 49–51, 54–56]. The variation between studies is likely due to differences in age and disease status of the sampled animals, farm type, sample type and testing method [57]. In agreement with this, the isolation frequency for *P. multocida* varied substantially between farms and calf groups in our study, as previously observed by others [29, 31, 51]. This could be due to differences in disease status between farms and calf groups, as discussed above, but in contrast to previous reports [29, 33], there was no statistically significant difference in isolation frequency for *P. multocida* between calves considered healthy and calves suspected for BRD in our study. This could be due to misclassification of calves as healthy or suspect of BRD since in contrast to the studies cited above [29, 33] we did not use an elaborate scoring system to classify calves.

Overgrowth of contaminants and mixed cultures from DNS were common in our study (data not shown) and made it difficult to recognise relevant bacterial pathogens in some samples which could lead to underdiagnosis. This was identified as problematic also by others [29] and is challenging when the aim is to obtain relevant bacterial isolates for susceptibility testing. It has been suggested that this could be partly overcome by cleaning the calves’ nares prior to sampling but also by efforts in the laboratory to identify relevant pathogens in mixed cultures [28]. On submission of samples, it might therefore be important to inform the laboratory that the main aim is not to identify specific infections in individual animals but to obtain relevant isolates for susceptibility testing representative of the group or farm.

Most TBL samples (61.6%) yielded no bacterial growth, but *P. multocida* was isolated from 22.4% of the samples and from at least one calf at 31 (73.8%) of the 42 sampling times of calf groups. This is within the range of results (5–44%) reported for healthy calves sampled by BAL or TTW [29, 33, 52, 58]. Our data are also in agreement with the isolation frequencies for calves with BRD sampled with the same TBL technique as in our study (16–34%) [59, 60] and in studies using BAL (21–30%) [29, 36]. Higher isolation frequencies in calves with BRD (40–80%) were obtained in other studies using BAL or TTW [28, 33, 50, 52].

There was no statistically significant difference in isolation frequency for *P. multocida* between calves considered healthy (20.2%) and those suspected for BRD (25.6%). However, several studies have reported that *P. multocida* is more often isolated from the lower respiratory tract of diseased calves [29, 33, 58, 60], although others have found no difference [52]. The lack of a statistically significant difference in our study could be due to misclassification of calves, as discussed above, and that it was not possible to determine the severity and stage of disease. The likelihood to isolate bacterial pathogens from the lower respiratory tract is influenced by the timing of sample collection and is probably higher after the acute stage has passed [5]. It should also be noted that marked respiratory disease can be caused by viral infections and occur without bacterial superinfection of the lower respiratory tract [29]. Moreover, it is likely that the sampling method influences the isolation frequency for bacterial pathogens. Endoscope guided BAL has been considered to give higher isolation frequencies than unguided BAL or TBL because affected lung lobes can be specifically sampled whereas a random part of the lower respiratory tract is sampled with the latter techniques [29, 36]. It is also suggested that the volume of fluid instilled is important [60]. A large, instilled volume probably increases the likelihood to isolate bacteria from the lower respiratory tract. This is corroborated by a generally higher isolation frequency in studies using TTW or BAL, where volumes of 50–180 mL fluid were instilled [28, 33, 50, 52], than in studies where smaller volumes were used [29, 59, 60]. In our study, we instilled 20 mL of isotonic saline with a mean retraction volume of 4.5 mL, and it is likely that the number of positive TBL samples would have been greater if a larger volume had been used.

In our study, ST-68 was the most common *P. multocida* ST found and the first report of this ST in farm animals. The second most common ST was ST-3, which was previously found in pigs from China [61] and in association with porcine pneumonia in Spain together with ST-19 [62], the third most common ST in our collection.

Knowledge of respiratory pathogens involved in an outbreak of BRD is important as a guide for treatment of animals and for disease management measures on a farm. However, there is a multitude of viral and bacterial pathogens involved [57] and even the epidemiology of *P. multocida* within a farm is complex [63]. For practitioners it is not possible to identify all pathogens, but to guide antibiotic therapy in a group of calves it may be sufficient to obtain a few representative isolates of the bacterial respiratory pathogens present and test them for antibiotic susceptibility. To this end, the practitioner has two options, either to sample the upper respiratory tract or the lower respiratory tract of a reasonable number of calves [36]. Sampling the lower respiratory tract has been considered more cost effective and appropriate in practice because samples are less contaminated, and the interpretation of culture results is therefore more straightforward [29]. Also, isolates from the lower respiratory tract are considered more relevant for identifying the pathogen causing infection in individual calves [5, 18, 32]. However, for practitioners, it is undoubtedly easier to collect samples from the upper respiratory tract, which is also considered less harmful for the animals [25, 31]. The risk of failure to isolate relevant pathogens from the upper respiratory tract in a group of calves can be minimised by sampling a larger number of animals, as previously suggested [15]. Our study showed that *P. multocida* isolated from upper respiratory tract samples are generally representative of isolates obtained from the lower respiratory tract of the same calf. This finding agrees with several other studies [15, 28, 33, 35, 36], and susceptibility testing of bacterial isolates collected by NPS or DNS from diseased calves was considered to yield relevant results for individual animals or calf groups [33, 35, 64].

Neither sampling the upper nor the lower respiratory tract would overcome the possibility that there can be several different *P. multocida* strains on a farm. Regardless of the sampling site, the culture results should therefore be interpreted with caution [36]. To reduce the risk of missing strains of the same bacterial species with different antibiotic susceptibility, several isolates could be selected on culture, but this approach might not be possible or cost effective in a clinical context.

Our study has several limitations. First, the isolates were collected between 1997 and 2000, and data regarding the genetic identity and STs of *P. multocida* and the occurrence of specific respiratory pathogens could be irrelevant for the current situation in Sweden. Moreover, the data and bacterial isolates are from clinical investigations on farms with BRD outbreaks, and sampling and bacteriological cultures were performed to guide practitioners in managing the outbreaks and not in the context of an experimental study. Therefore, the processing of samples in the laboratory was performed as part of the routine work and focused on identifying *Pasteurellacae*. More elaborate efforts to identify isolates in the samples were not made, and only one isolate from each sample was further evaluated and saved. This probably led to underdiagnosis of bacterial pathogens in both DNS and TBL. Another limitation is that *Mycoplasmopsis* spp. were not cultured because the methodology was not available at the laboratory at the time of the field investigations. Further, although all isolates were tested for antibiotic susceptibility, the method and interpretive criteria used at that time are largely obsolete and the data were considered unreliable and therefore not included in the study. The lack of susceptibility data precludes evaluation of the possibility that there could be mobile genetic elements carrying resistance genes in one of the *P. multocida* isolates in an isolate pair that were considered identical after cgMLST. This would lead to differences in antibiotic susceptibility between the DNS and TBL isolate. Despite these limitations, we believe that the evaluation of DNS in relation to TBL and conclusions regarding the sampling of calves might be relevant for BRD diagnosis and management.

## Conclusions

In this study, *P. multocida* was readily isolated from the upper airways of calves. Although there was a large variation in its isolation frequency between farms and calf groups, at least one *P. multocida* isolate was obtained from DNS in 83.3% of the calf groups on 25 farms. It was found that in 75% of the calves where *P. multocida* was isolated from DNS and TBL isolates interpreted as identical by cgMLST were obtained by both sampling methods. During an outbreak of BRD in a calf group, a simple approach for practitioners to gain insight in the presence of *P. multocida* in the lower respiratory tract could be to collect and submit samples from the upper respiratory tract of 4–6 calves within the group.

## Supplementary Information

Additional file 1.

Additional file 2.

## Data Availability

The datasets used and analysed during the current study are available from the corresponding author on reasonable request.

## Declarations

### Prior publication

Data have not been published previously.
